# Supplementary material for: SNAP timing and food insecurity
Source: PLoS One. 2021 Feb 25;16(2):e0246946. doi: 10.1371/journal.pone.0246946 (PMC7906471; doi:10.1371/journal.pone.0246946)
Supplement: S1 Appendix — (DOCX) [file pone.0246946.s001.docx]

**S1 Appendix. Linear Regression Parameters.**

| VARIABLES | RunOut | NotLast | BalMeal | CutSkip | CutSkipF | EatLess | Hungry | LoseWgt | WholeDay | WholeDayF |
| --- | --- | --- | --- | --- | --- | --- | --- | --- | --- | --- |
| Sample Person Characteristics: |  |  |  |  |  |  |  |  |  |  |
| Male | 0.0180 | 0.00898 | 0.0271* | 0.0278** | 0.0135 | 0.0223* | 0.0218* | 0.00830 | 0.00378 | 0.00410 |
|  | (0.0122) | (0.0146) | (0.0145) | (0.0135) | (0.0112) | (0.0123) | (0.0118) | (0.00735) | (0.00745) | (0.00727) |
| Age | -5.53e-05 | 0.000356 | 0.000774 | -0.000548 | -5.08e-05 | -0.000464 | -0.000364 | 0.000101 | 3.94e-05 | -3.25e-05 |
|  | (0.000481) | (0.000574) | (0.000513) | (0.000456) | (0.000421) | (0.000431) | (0.000367) | (0.000237) | (0.000251) | (0.000227) |
| Hispanic | 0.0738** | 0.0842** | 0.0498 | -0.0383 | -0.0172 | -0.0520* | -0.0447* | -0.0162 | -0.0433** | -0.0376** |
|  | (0.0360) | (0.0400) | (0.0332) | (0.0320) | (0.0283) | (0.0305) | (0.0248) | (0.0163) | (0.0175) | (0.0164) |
| Black | -0.00545 | -0.00511 | -0.0786*** | -0.0747** | -0.0477* | -0.0819*** | -0.0370 | 0.0230 | -0.00814 | -0.0116 |
|  | (0.0343) | (0.0394) | (0.0290) | (0.0282) | (0.0273) | (0.0299) | (0.0296) | (0.0189) | (0.0197) | (0.0190) |
| Other Race | 0.0516 | 0.0129 | 0.0491 | -0.0142 | 0.00677 | 0.0229 | 0.0252 | 0.0368 | -0.0124 | 0.000669 |
|  | (0.0471) | (0.0601) | (0.0501) | (0.0456) | (0.0449) | (0.0488) | (0.0469) | (0.0305) | (0.0422) | (0.0404) |
| Household Characteristics |  |  |  |  |  |  |  |  |  |  |
| Family Size | 0.0147* | 0.0128* | 0.00382 | -0.00643 | -0.00449 | -0.00117 | -0.00496 | -0.00863** | -0.00273 | -0.00210 |
|  | (0.00811) | (0.00755) | (0.00746) | (0.00782) | (0.00840) | (0.00806) | (0.00712) | (0.00404) | (0.00643) | (0.00670) |
| Poverty-to-Income Ratio | -0.0423 | -0.0331 | -0.0200 | 0.0296 | -0.0112 | 0.0203 | 0.0309 | -0.0166* | -0.0212* | -0.0208* |
|  | (0.0288) | (0.0346) | (0.0359) | (0.0345) | (0.0194) | (0.0364) | (0.0384) | (0.00970) | (0.0119) | (0.0107) |
| Ref Person Married | 0.00197 | -0.0170 | -0.0124 | -0.0173 | -0.0115 | -0.0133 | -0.00726 | 0.0140 | -0.00352 | -0.00655 |
|  | (0.0300) | (0.0341) | (0.0338) | (0.0316) | (0.0299) | (0.0357) | (0.0295) | (0.0171) | (0.0191) | (0.0202) |
| Ref Person HS Grad | 0.0191 | -0.0440 | -0.0775*** | -0.0210 | 0.000825 | -0.00517 | -0.0351 | -0.0119 | 0.000814 | -0.00268 |
|  | (0.0291) | (0.0283) | (0.0291) | (0.0255) | (0.0233) | (0.0322) | (0.0235) | (0.0152) | (0.0159) | (0.0162) |
| Ref Person Some College | -0.0167 | -0.0438 | -0.0659** | -0.0272 | 0.00213 | -0.00739 | -0.00503 | -0.00281 | 0.00389 | -0.00105 |
|  | (0.0301) | (0.0318) | (0.0298) | (0.0278) | (0.0240) | (0.0260) | (0.0202) | (0.0131) | (0.0168) | (0.0171) |
| Ref Person College Grad | -0.0133 | -0.115* | -0.101 | -0.0851* | -0.0355 | -0.124** | -0.0908** | 0.00881 | -0.0654*** | -0.0488*** |
|  | (0.0677) | (0.0649) | (0.0682) | (0.0496) | (0.0540) | (0.0467) | (0.0409) | (0.0400) | (0.0149) | (0.0155) |
| $2000 Liquid Assets | -0.341*** | -0.278*** | -0.159** | -0.147*** | -0.121*** | -0.157*** | -0.109*** | -0.0666*** | -0.00945 | -0.0210 |
|  | (0.0596) | (0.0585) | (0.0622) | (0.0397) | (0.0312) | (0.0433) | (0.0254) | (0.0150) | (0.0208) | (0.0170) |
| Participate in WIC | -0.0422 | -0.0517* | -0.0454* | -0.0459** | -0.0477*** | -0.0419** | -0.0123 | -0.00102 | -0.0112 | -0.0171 |
|  | (0.0287) | (0.0284) | (0.0255) | (0.0191) | (0.0170) | (0.0202) | (0.0149) | (0.0118) | (0.0130) | (0.0105) |
| In Salience Window | 0.00348 | -0.000912 | 0.0298 | 0.0551*** | 0.0312* | 0.0614*** | 0.0441** | 0.0271** | 0.0451*** | 0.0420*** |
|  | (0.0231) | (0.0205) | (0.0255) | (0.0177) | (0.0179) | (0.0204) | (0.0185) | (0.0133) | (0.0154) | (0.0145) |
| Constant | 0.549*** | 0.468*** | 0.373*** | 0.249*** | 0.197*** | 0.256*** | 0.135*** | 0.0952*** | 0.0796** | 0.0663* |
|  | (0.0454) | (0.0533) | (0.0567) | (0.0470) | (0.0463) | (0.0470) | (0.0389) | (0.0273) | (0.0337) | (0.0345) |
|  |  |  |  |  |  |  |  |  |  |  |
| Observations | 7,919 | | | | | | | | | |
| R-squared | 0.035 | 0.031 | 0.028 | 0.026 | 0.018 | 0.025 | 0.027 | 0.016 | 0.024 | 0.024 |
