# Supplementary material for: SNAP timing and food insecurity
Source: PLoS One. 2021 Feb 25;16(2):e0246946. doi: 10.1371/journal.pone.0246946 (PMC7906471; doi:10.1371/journal.pone.0246946)
Supplement: Attachment — Submitted filename: PlosONE Review 1.docx [file pone.0246946.s002.docx]

PONE-D-20-32057_review

SNAP Timing and Food Insecurity

There has been some really cool research done using the variation in SNAP distribution dates within and across states within the last 5 years or so. SNAP receipt unlike other benefits is not perfectly aligned with timing of other income and is distributed over one to almost 20 days within a month depending on the state, according to a random case number assigned or using the last name of the recipient etc. Everyone receives it once a month but at different times within a calendar month according to the schedule adopted by the state, they live in.

The variation in timing and changes in the level of food security over the course of the benefit month has been studied as a mechanism in many of the above-mentioned work (though I am not giving any citations here, many of them are mentioned in this manuscript) and it is nice to see a paper exploring this mechanism as the outcome of interest. Though the paper is not about SNAP and food insecurity; It is an attempt to better understand the extend of food insecurity. By utilizing response difference in two sets of food insecurity questions (a 30-day recall versus a 12-month recall questionnaire) and differences in receipt timing relative to the interview date they try to gauge the extend of possible underestimation of food insecurity and additional underestimation of SNAP’s effectiveness in reducing this food insecurity due to salience of food insecurity, some behavioral reasons such like framing.

First of all, I think it is an interesting concept to study. Their way of trying to get at reporting differences as a function of salience of the benefit receipt is also a worthy endeavor. I think their econometric work is very solid.

My biggest problem is that there are too many things going on in the paper. It is hard to understand even for someone like me who is familiar with the literature. They need to do a better job stating their question/goal and maybe trim some of the analysis to an online supplement.

Here are my other issues:

Unlike other papers using SNAP timing they do not utilize the differences across states’ distribution schedules. Which can actually provide a nice dimension since in some states it is more uniformly distributed over the month than the others. It will be also nice to compare states where the SNAP month overlaps with the calendar month or other payment schedules and states where it is not.

The group for which they have data for 30-day and 12-month questions are cohorts from 2007-2008 and 2009 -2010. Can this be the source of the differences in what they see? These cohorts may have marginal new receivers of food stamps (there were many new entrants due to Great Recession) who may be experiencing food insecurity very differently. They may actually be not on food stamps over the course of the last year and had experience food insecurity unlike anything before and may have very different recall of the initial shock compared to their recent food insecurity experiences. Not sure if this is the case as I did not have time to think this through but if they have the data it may be really good to see if people who are recent SNAP recipients experience food insecurity SNAP schedule differently than long time receivers.

Salience window x-axis not sure what they capture in Figure 3 compared to other figures salience window axis. Do they have the same interpretation as the axis in later figures with salience windows, defined in days?

It is interesting that the 12-month recall correlates with the snap cycle. Is 30-day FS survey always after the 12-month survey?

Also, NHANES has some consumption data or some data on what they ate recently /nutrients in their body (I am not very familiar with this data set)? If this is indeed the case can we use this as a way to get it objective or subjective (framing) food insecurity? It may be interesting to look at nutrient deficiencies of 30-day vs 12-month VLFS people. Both within the group that has both surveys and for all 12-month respondents to address my earlier issue. This may be off target though and if so this again shows the paper is hard to follow at times.

Page 5 (second from the last line) should it be “food insecurity” and not “food security”

There are some squares in the text (looks like they are replacing long dashes)
